# Raising active children: how family and school shape health-promoting physical activity—findings from the FAMIPASS study

**DOI:** 10.3389/fspor.2025.1530398

**Published:** 2025-02-07

**Authors:** Michal Vorlíček, Jan Dygrýn, David Janda, Jaroslava Voráčová, Scott Duncan, Erik Sigmund, Dagmar Sigmundová

**Affiliations:** ^1^ Institute of Active Lifestyle, Faculty of Physical Culture, Palacký University Olomouc, Olomouc, Czechia; ^2^ Department of Natural Sciences in Kinanthropology, Faculty of Physical Culture, Palacký University Olomouc, Olomouc, Czechia; ^3^ Department of Social Sciences in Kinanthropology, Faculty of Physical Culture, Palacký University Olomouc, Olomouc, Czechia; ^4^ Human Potential Centre, School of Sport and Recreation, Auckland University of Technology, Auckland, New Zealand

**Keywords:** movement behaviors, recommendations for physical activity, accelerometry, family characteristics, child, preschool

## Abstract

This study investigated the combined impact of family dynamics and school environments on physical activity levels in children aged 3–9 years across distinct segments of the school day. Conducted as part of the FAMIPASS project in the Czech Republic, the study collected data in 2022 and 2023 from 502 families affiliated with 36 preschools and primary schools. The device-based monitoring of movement behaviors in children and their parents was conducted over a one-week period using ActiGraph accelerometers, complemented by detailed family questionnaires. Regression analysis revealed that parental physical activity, BMI, and education level significantly influenced children's moderate-to-vigorous physical activity, with educated parents more likely to raise active children. Active transport to school emerged as a key factor associated with higher child activity levels specifically in the time segment before school. This research underscores the role of family and school as critical arenas for promoting health and physical activity. These insights highlight the need for integrated family-school strategies to foster healthy activity habits in children, thereby laying the groundwork for a more active generation.

## Introduction

Regular movement behaviors, including physical activity (PA), are essential for the healthy development of children and young people (1, 2). Numerous studies have shown that regular PA improves not only physical fitness (3) but also cardiometabolic health (4), bone density (5), mental health (6), and cognitive functions (3, 7). Moderate-to-vigorous physical activity (MVPA), characterized by activities that elevate heart rate and breathing (e.g., brisk walking, running, cycling), is particularly emphasized in global recommendations. According to WHO guidelines, children aged 5–17 should engage in at least 60 min of MVPA daily to support their physical and mental well-being (8). Despite these benefits, there is a growing concern about the prevalence of sedentary behaviors (SB) worldwide (9, 10). Technology-driven societal change, accelerated by the COVID-19 pandemic (11), leads to a reduction in PA, with many children failing to meet the global movement behavior recommendations (12, 13).

The family and school environments are the two most important influences on children's movement behaviors (14). Parents play a crucial role in modeling active behaviors and promoting active transport, which significantly increases children's overall PA levels (15). Research has indicated that parental involvement, including encouragement and participation in PAs, is a key predictor of children's movement behaviors (16). Additionally, even parents who may not engage in PAs themselves can positively impact their children's activity levels through supportive behaviors (17, 18). This suggests that active role models within the family can foster a culture of PA among children.

Concurrently, the influence of kindergarten/school-based PA opportunities is also vital (19, 20). Carlson et al. noted that socioeconomic disparities in school practices can affect children's PA levels during school hours, emphasizing the role of schools in providing structured opportunities for movement (21). Comprehensive whole-school PA programs within schools can enhance children's activity levels (22–24), particularly in low-income settings with limited resources (25). Furthermore, the interplay between family dynamics and school-based opportunities is crucial for understanding children's movement behaviors throughout the day. Unfortunately, most studies tend to examine these two domains in isolation, limiting our understanding of how family and school factors collectively influence children's PA patterns.

The FAMIPASS study (FAMIly Physical Activity, Sedentary Behavior, and Sleep) addressed this gap by utilizing objective data from accelerometers to assess movement behaviors in children (26). An important feature of this study included the segmentation of the day into three parts: before, during and after school/kindergarten. This allowed for a nuanced analysis of how school environments influence PA, both directly through structured activities and indirectly through family-related factors such as parental education and PA levels. Additionally, unlike many previous studies that separately assessed either parents' or children's activity, the FAMIPASS study collected synchronized data from entire families. This enables a comprehensive analysis of parent-child associations in movement behaviors, contributing valuable insights into how the family unit operates in relation to PA. By examining these dynamics across distinct time segments of the day, the study provided a robust framework for understanding the role of both schools and families in promoting children's movement behaviors.

The purpose of this manuscript was to present the results of the FAMIPASS study, with a particular focus on the role of schools in supporting children's PA throughout different segments of the school day, and how family factors, particularly parental education, influence these patterns. This research was aimed to offer evidence-based insights into the interplay between school environments, family characteristics, and children's movement behaviors, contributing to the broader goal of developing sustainable strategies to increase PA levels among young people.

## Method

### FAMIPASS study

The FAMIPASS study is a cross-sectional investigation of 24 h movement behaviors among families with young children. In this context, a family is characterized as a unit comprising one or more parents and their children cohabiting in the same household. Recruitment of participating families was conducted through preschools and primary schools selected from a stratified sample of urban and rural regions across Bohemia, Moravia, and Silesia. Measurements of both children and parents were taken within their natural daily routines and environments (26).

### Participants and dataset

Anthropometric data for all family members, along with socioeconomic and demographic characteristics, were collected using a family diary and a questionnaire completed by the parents. Between March 2022 and May 2023 (no COVID-19 restrictions have been applied), 24 h movement behaviors, including PA, SB, and sleep, were continuously monitored with wrist-worn accelerometers over a 7-day period during a regular kindergarten or school week, avoiding multi-day vacations and holidays. Inclusion in the study required that at least one parent participate and that the following criteria be met: (a) the child's age fell within the range of 3–8 years (siblings can also participate); (b) all participants were in good health, without any medical conditions that would prevent them from engaging in typical activities at school, kindergarten, or work; and (c) all participants agreed to participate voluntarily and without financial compensation (26). Out of the 860 families contacted, 552 provided signed informed consent to participate. Of these, 502 families began the 7-day period of continuous movement behavior monitoring, and 472 families completed it successfully. Participants were excluded from the final dataset for missing anthropometric data, missing gender information, or insufficient data to calculate family socioeconomic status, resulting in a further 26 exclusions. For the purpose of this sub-study, only participants with a flawlessly completed daily log were included, resulting in a final sample of 249 children (51.8% girls). Along with these children, 238 mothers and 197 fathers participated in the study (Table 1).

**Table 1:** Descriptives.

	Median	IQR
Gender (% Girls)	51.8	
Age, years	6.6	2.58
BMI *z*-score	−0.10	1.40
Age (fathers)	39.5	7.00
BMI (fathers)	26.0	4.99
Age (mothers)	36.4	4.88
BMI (mothers)	23.4	3.98

Abbreviations: IQR, interquartile range.

Researchers instructed parents on wearing the accelerometer and recording 24 h movement behavior in a family diary. Organized in a table format, the diary allowed parents to conveniently log times related to daily routines on school days and weekends, including wake-up time, arrival and departure from kindergarten or school, physical education classes, organized PAs (e.g., training sessions, clubs), bedtime, and transport modes (e.g., walking, cycling, car, public transport). The 24 h movement monitoring with the accelerometer began at midnight on the day of the instructional meeting, during which parents were directed to place the device on themselves and their children before bed. After the 7-day monitoring period, researchers collected accelerometers and completed diaries.

### Measurement of movement behaviors

Children's and parents' 24 h movement behaviors were continuously monitored over seven days using accelerometers worn on the wrist of the non-dominant hand, except during bathing and swimming. ActiGraph wGT3X-BT accelerometers were used for children, while GT9X Link devices were provided for parents (both devices from ActiGraph LLC, Pensacola, FL, USA). Wrist-worn accelerometers have been shown to have higher wear-time compliance and acceptability compared with hip-worn devices (27)and to reliably capture activity during activities of daily living (28) Each device was individually initialized for each family member with ActiLife software version 6.13.4 (ActiGraph LLC, Pensacola, FL, USA), configured to record triaxial acceleration data at 100 Hz. To prevent mix-ups among family members, each accelerometer was labeled and specifically assigned to an individual. Accelerometer data was processed using the open-source R package GGIR version 2.7–1 (29), with previously validated thresholds to classify category of movement behaviors. SB corresponded to acceleration values below 36 m*g*, light PA range from 36 to 200 m*g*, MVPA at 201 m*g* or higher (30, 31).

### Segments of the school days

We performed activity-log-based segmentation using the R package GGIR (version 2.7–1). The segment “Before school” was defined as the time between wake-up and the starting time of school. The segment “During school” was defined as the time between the start and end of school. Finally, the “After school” time segment was defined as the time between the end of school and sleep onset. Only days with start and end times recorded by parents, as well as valid accelerometer-identified times for wake-up and sleep onset, were analyzed. A valid accelerometer day was defined as having at least 75% wear time during both the wake-up period and sleep time. Only participants with at least three valid school days were included in the final analysis (32).

### Predictors and potential covariates

The informed consent included questions for parents regarding the collection of participants' anthropometric data. Prior to the start of the 24 h movement behavior monitoring, parents answered simple questions about basic anthropometric parameters (gender, month and year of birth, and body height/weight to the nearest 0.5 cm/0.1 kg) for each participating family member at home. Using weight and height measurements performed by parents at home following standardized protocols—which have been validated as sufficiently accurate for calculating body mass index (BMI) in children (33) we calculated BMI z-scores for each child according to age- and gender-specific reference values from the World Health Organization (34).

Six questions about families' material wealth from the Family Affluence Scale, which were part of the family diary, were used to assess family SES. The original questions used to determine SES were obtained from the international research study named Health Behavior in School-aged Children, then translated, validated, and reused in previous studies including Czech families with children (26, 35). The content of the six questions and their response options were as follows: having one's own bedroom for each child in the family (0 or 1); number of computers in the household (0, 1, 2 and ≥3); number of cars owned for family use (0, 1 and ≥2); number of foreign holidays taken in the past year (0, 1, 2 and ≥3); ownership of a dishwasher (0 or 1); number of bathrooms in the household (0, 1, 2 and ≥3). The sum of all six questions formed a summary score from which three categories of family SES were calculated as follows: the lowest/highest 20% of the summary score characterized families with “low”/“high” SES, and the range of 21%–79% of the summary score identified families with “medium” SES (26, 35).

Children were classified as actively commuting to school if they reported an active mode of transport (walking, cycling, or using a scooter) on the majority of school days. We defined a safe neighborhood environment as one where parents agreed with the statement: “The neighborhood is safe for children to walk or play outside during the day”.

### The ethics

The study was approved by the Ethics Committee of the Faculty of Physical Culture at Palacký University Olomouc on 28 February 2021, ref. no. 25/2021. The approval from the Ethics Committee included the methodological protocol, which contained the conditions of participation in the research as stated in the informed consent for parents and their offspring. The techniques and instruments used were health- and hygiene-compliant and were disinfected before each use. Personal information was anonymized, as were responses on SES and the background of the families' environment. None of the participants were penalized in case of damage or loss of the accelerometer or interruption or non-completion of the research. Participation in the research was voluntary and free of charge. Each research participant received individualized feedback on the results of monitoring his or her own 24 h movement behavior in the form of graphic sheets with explanatory commentary. The overall results of the study for the kindergarten/school were compiled for the representatives of the participating schools, together with a thank you note and a certificate of participation in the research.

### Statistical analysis

The analysis was conducted using R statistical software version 4.4.1. (36). Normality of continuous variables was assessed using visual inspection of histograms and formal testing using the Shapiro–Wilk tests. Due to the violation of normality (Shapiro–Wilk test *p* < 0.05) in some of our variables of interested the descriptive statistics were presented as median and interquartile range (IQR) for continuous variables and as percentages for categorical variables. The level of statistical significance was set at α < 0.05.

Due to the sparseness of our dataset, we decided to use multiple imputations for variables with less than 10% missing data. This decision was made to ensure that we did not predict a larger proportion of our variables of interest, such as father's BMI (21.7% missing), father's education (25.3% missing), mother's MVPA (47.8% missing), and father's MVPA (61.5% missing). When these variables were included in the analyses, we used list-wise deletion. Variables with less than 10% missing data were imputed under the assumption of missing completely at random using predictive mean matching for children's BMI z-score (*n* = 2) and mother's BMI (*n* = 14). The logistic regression method was used for active transport to school (*n* = 2), type of residence (*n* = 7), safety (*n* = 8), and maternal education (*n* = 16). A proportional odds model was used to impute Family Affluence Scale categories (*n* = 6). We used the default settings of the mice package (37), with 5 iterations to create 5 imputed datasets, utilizing the rest of the variables in the dataset as predictors. To evaluate the robustness of our multiple imputation approach, we performed a complete-case analysis that excluded all individuals with missing data on the variables of interest. We then re-estimated the same models on this reduced dataset and compared the parameter estimates, confidence intervals, and significance levels to those from the multiply imputed data. The results were broadly consistent, suggesting that the conclusions drawn from our primary (imputed) dataset were not unduly influenced by the imputation process.

The associations between children's MVPA during different segments of the school day (dependent variable) and family characteristics (independent variables) were analyzed using multivariate mixed-effect linear regression. We specified a two-level structure in which Level 1 were the characteristics of each family and Level 2 was the location of the data collection (i.e., the cluster). Mixed-effects models were chosen due to an improvement in model fit when including the location of data collection as a random effect, accounting for potential clustering of observations within locations.

A total of nine mixed-effects models were constructed, corresponding to the combinations of the three daily segments (before, during, and after school) and the three groups of characteristics (maternal, paternal and contextual factors). The contextual characteristics included type of residence (house/flat), Family Affluence Score categories (low/medium/high), perceived safety (yes/no), and active transport to school (yes/no; used only for the segment before school). The maternal characteristics included BMI, education (university/lower), and MVPA of mothers. The paternal characteristics included BMI, education (university/lower), and MVPA of fathers. All models were controlled for child characteristics including age, gender and BMI *z*-score. The variance inflation factor (VIF < 2 for all models) indicated no multicollinearity issue. Analyses were performed using the “lme4” package in R (38). All models were adjusted for potential covariates, including children's age, gender, and BMI *z*-score.

## Results

The following section provides a detailed description of the average level of MVPA observed in children during three segments of the school day: before school, during school hours, and after school. The median duration of MVPA before school was 2.69 min (IQR = 3.38), while during school hours it was 40.25 min (IQR = 24.84). After school, children exhibited a median of 37.45 min of MVPA (IQR = 24.49). The duration of each segment also varied, with the before-school segment median of 62.20 min (IQR = 31.71), the school segment 413.27 min (SD = 94.92), and the after-school segment 549.35 min (SD = 90.37). The results indicated that most PA occurred during and after school, with relatively low activity levels in the morning (Table 2).

**Table 2:** Description of MVPA of children per segment.

	Median	IQR	Minimum	Maximum
MPVA before school (min/day)	2.69	3.38	0.00	17.42
MVPA during school (min/day)	40.25	24.84	4.67	102.55
MVPA after school (min/day)	37.45	24.49	5.32	118.15
Segment duration before school (min/day)	62.20	31.71	9.33	170.61
Segment duration during school (min/day)	413.27	94.92	220.13	661.91
Segment duration after school (min/day)	549.35	90.37	340.39	741.50
MVPA before school/MVPA total (%)	3.42	3.60	0.00	23.89
MVPA before school/duration of the segment (%)	4.72	4.85	0.00	27.28
MVPA during school/MVPA total (%)	47.85	18.04	11.92	83.70
MVPA during school/duration of the segment (%)	9.54	5.63	1.90	21.68
MVPA after school/MVPA total (%)	47.39	19.45	10.73	80.54
MVPA after school/duration of the segment (%)	7.05	4.17	1.17	19.46

Abbreviations: IQR, interquartile range; MVPA, moderate-to-vigorous physical activity.

The ratios of segmental MVPA to total MVPA (per whole day) and to the duration of each segment are presented in Table 2. At the median, MVPA before school accounted for 3.42% (IQR = 3.60) of the total MVPA and 4.72% (IQR = 4.85) of the duration of the segment. During school hours, MVPA represented 47.85% (IQR = 18.04) of the total MVPA and 9.54% (IQR = 5.63) of the duration of the segment. After school, MVPA contributed to 47.39% (IQR = 19.45) of the total MVPA and 7.05% (IQR = 4.17) of the duration of the segment. Table 2 shows that while the majority of MVPA occurred during and after school, only about 3% of the before-school time segment was spent in MVPA, indicating relatively low PA levels compared to the other parts of the day.

Table 3 summarizes the associations between parental indicators and children's MVPA during different segments of the school day. The results showed that maternal BMI had no significant association with children's MVPA before school (*B* = 0.00, *p* = 0.991) but was negatively associated during school hours (*B* = −0.15, *p* = 0.031). There was no significant association between maternal BMI and children's MVPA after school. Maternal education at the university level showed a trend towards a negative association with children's MVPA during school hours (*B* = −1.19, *p* = 0.066). Maternal MVPA showed no significant association with children's MVPA before school but indicated a weak positive association during and after school, although these associations did not reach statistical significance.

**Table 3:** Association between parental indicators and MVPA per segment.

	Before school MVPA/duration of the segment			In school MVPA/duration of the segment			After school MVPA/duration of the segment		
	*B*	95% CI	*p*-value	*B*	95% CI	*p*-value	*B*	95% CI	*p*-value
Model 1—Maternal indicators (*n* = 130)^b^									
Mother BMI	0.00	−0.12; 0.11	0.991	**−0** **.** **15**	**−0.28; −0.01**	**0** **.** **031**	0.02	−0.10; 0.14	0.740
Mother education (University)^a^	0.58	−0.44; 1.60	0.279	−1.19	−2.46; 0.09	0.066	−0.19	−1.31; 0.93	0.734
Mother MVPA	0.00	0.00; 0.01	0.325	0.01	0.00; 0.02	0.171	0.01	0.00; 0.02	0.060
Model 2—Paternal indicators (*n* = 89)^c^									
Father BMI	**−0** **.** **28**	**−0.49; −0.07**	**0** **.** **010**	0.12	−0.06; 0.29	0.178	0.06	−0.10; 0.22	0.485
Father education (University)^a^	−0.13	−2.00; 1.73	0.887	−0.50	−2.07; 1.07	0.528	**1** **.** **45**	**0.00; 2.90**	**0** **.** **049**
Father MVPA	0.00	−0.01; 0.02	0.850	**0** **.** **02**	**0.01; 0.03**	**<0** **.** **001**	**0** **.** **01**	**0.00; 0.02**	**0** **.** **046**

Boldface values indicate significant association at *p* < 0.05.

^a^ Reference value was set to Education = lower than university degree.

^b^ Models were adjusted for children's age, sex, body mass index *z*-score, and body mass index, education, and moderate-to-vigorous physical activity of mothers.

^c^ Models were adjusted for children's age, sex, body mass index *z*-score, and body mass index, education, and moderate-to-vigorous physical activity of fathers.

Paternal BMI was significantly negatively associated with children's MVPA before school (*B* = −0.28, *p* = 0.010), while no significant associations were observed during or after school. Paternal education at the university level showed a significant positive association with children's MVPA after school (*B* = 1.45, *p* = 0.049). Paternal MVPA was positively associated with children's MVPA during and after school, with the strongest association observed during school hours (*B* = 0.02, *p* < 0.001). These findings suggest that parental PA, particularly that of fathers, plays an important role in influencing children's PA levels during and after school hours (Table 3).

The sample was characterized by a high perception of neighborhood safety, with 86% of parents considering their area safe for children's PA and play during the day. Socioeconomic status, as measured by FAS, showed that 13% of families were categorized as low, 67% as middle, and 20% as high. Regarding housing, 65% of families lived in detached houses, 22% in apartment buildings, and 14% in panel housing. Active transport to school was reported by 41% of children. Associations between these contextual factors and MVPA during each segment of the school day are presented in Table 4. Active transport was significantly positively associated with MVPA before school (*B* = 1.90, *p* < 0.001), suggesting that children using active modes of transport, such as walking or cycling, were more likely to be physically active during this segment. However, no significant associations were found between contextual factors and MVPA during or after school.

**Table 4:** Association between contextual factors and MVPA per segment.

	Before school MVPA/duration of the segment			In school MVPA/duration of the segment			After school MVPA/duration of the segment		
	*B*	95% CI	*p*-value	*B*	95% CI	*p*-value	*B*	95% CI	*p*-value
Model 3—Contextual factors (*n* = 249)									
Residence type (House)^a^	−0.52	−1.61; 0.58	0.366	0.05	−1.09 1.19	0.932	−0.71	−1.68 0.26	0.148
FAS (Middle)^b^	0.73	−0.70; 2.17	0.327	1.40	−0.10 2.88	0.070	0.39	−0.86 1.65	0.536
FAS (High)^b^	1.38	−0.34; 3.07	0.119	1.10	−0.70 2.88	0.233	0.73	−0.77 2.23	0.337
Neighborhood safety (No)^c^	0.82	−0.51; 2.14	0.228	0.49	−0.91 1.86	0.486	−0.33	−1.48 0.83	0.579
Active transport (yes)^d^	**1** **.** **90**	**0.94; 2.85**	<0.001						

Boldface values indicate significant association at *p* < 0.05.

All models were additionally adjusted for children's age, sex, body mass index *z*-score.

CI, confidence intervals; FAS, family affluence scale; MVPA, moderate to vigorous physical activity.

^a^ Reference value was set to residence type = apartment.

^b^ Reference value was set to FAS = low.

^c^ Reference values was set to Safety = yes.

^d^ Reference value was set to Active transport = no.

## Discussion

The present study highlighted the critical role of schools in supporting children's PA throughout different segments of the school day. The findings demonstrated that the majority of MVPA occurred during and after school hours [as in the case of Czech adolescents (39]), with relatively low activity levels before school. These results emphasize the importance of providing structured opportunities for PA within the school environment, not only during physical education classes, but also through initiatives like active breaks (40, 41) and extracurricular activities (42). Schools can be pivotal in bridging the gap in PA levels by offering an environment conducive to movement, particularly for children who may have fewer opportunities to be active outside school hours (24, 43).

The role of active transport as a predictor of MVPA before school further underscores the potential of school-led initiatives to promote active commuting. Schools can support active commuting through initiatives like “walking buses” or bike-to-school campaigns, while municipalities can enhance these efforts by creating safe and accessible infrastructure, such as bike lanes or pedestrian zones near schools. City-supported programs, like “Safe Routes to School” (https://1url.cz/J1055) could further encourage active transport and collaboration between schools and local councils to foster neighborhood safety, making it easier for families to adopt active lifestyles. Encouraging walking or cycling to school, especially through campaigns or partnerships with local communities, could provide a feasible and effective strategy for increasing overall PA levels (44). The success of these initiatives, however, relies on safe infrastructure, such as sidewalks and bike lanes (45), and perceived neighborhood safety (46, 47). Collaboration between schools, city councils, and communities is essential to create supportive environments for active transport (48, 49). Schools and city councils that facilitate a safe and supportive environment for active commuting are likely to see benefits not only in increased MVPA but also in improved attention and readiness to learn in the classroom (50, 51). Integrating education about active transport into the school curriculum could also raise awareness among children and their families about the health benefits of walking and cycling, fostering lifelong healthy habits (52, 53).

A unique feature of this study was its focus on the family unit rather than solely on children. By involving parents and employing device-based monitoring of movement behaviors using ActiGraph accelerometers, this research provided a comprehensive picture of the dynamics that influence children's activity levels. The significant association observed between fathers’ PA and children's MVPA during school hours, as shown in our study, emphasizes the influential role of paternal behaviors in shaping children's PA patterns. This finding suggests that children may primarily model their PA habits particularly after their fathers, at least within the school-day context. Possible explanations include fathers' greater interest in sport in general (54) and their increased involvement as coaches or assistants in sports clubs, while mothers tend to focus more on enforcing sleep-related rules and limiting excessive sedentary behavior in their offspring (55). These gender-related differences in parent-child roles and behaviors could be seen as a potential limitation of the study, as they reflect societal norms that may not apply universally. Future research could explore how these dynamics vary across different cultural contexts.

However, when examining the entire FAMIPASS study sample, this association was significant for both fathers and mothers, and it extended not only to MVPA but also to overall PA (35). These findings align with previous FAMIPASS studies, which have demonstrated the critical role of maternal physical activity and sleep behaviors in supporting adherence to PA and sleep guidelines in children (35, 55). For example, reduced sedentary behavior and the absence of screen-time devices in children's bedrooms have been associated with better adherence to sleep recommendations (35). Moreover, significant associations have been identified between MVPA and total PA in parent-child pairs across all gender combinations, further emphasizing the importance of parental role modeling in fostering active behaviors. Additionally, it has been shown that overweight children exhibit significantly lower PA and higher SB compared to their non-overweight peers (56). These findings underscore the need for family-centered interventions targeting not only children but also parental behaviors, as such interventions may play a pivotal role in addressing both PA and sedentary behavior patterns within families. Interestingly, no significant differences were found in the prevalence of excess body weight in children based on age, gender, family SES, or parental obesity (56), highlighting the complexity of factors contributing to childhood overweight.These broader FAMIPASS results highlighted the potential value of family-centered, school-based programs that encourage active lifestyles across the entire family, with targeted interventions that address the unique contributions of both parents. In our view, schools can play a leading role in engaging parents through workshops, events, or family-oriented PA initiatives that extend beyond the traditional school setting (57).

Furthermore, the whole-day segments approach adopted in this study provided a more nuanced understanding of children's movement behaviors throughout the entire day. Such a comprehensive analysis is often absent in earlier studies, which focus mainly on school hours (58, 59) or after-school periods (60). This comprehensive approach reveals that while schools provide a structured environment that promotes PA, the influence of family dynamics cannot be underestimated (61, 62). The interaction between school policies, family behaviors, and children's PA patterns suggests that a holistic approach involving both schools and families is necessary to maximize the health benefits of PA (63–65).

The findings of the FAMIPASS study underscore the need for schools to play a proactive role in promoting PA across multiple domains—through in-school activities, support for active commuting, and family engagement. Future interventions should aim at enhancing school-based PA programs while also extending support to families to ensure that active lifestyles are fostered both at school and at home. Finally, while active transport has been identified as a significant contributor to MVPA, a rigorous analysis of Czech children's active transport to school is still lacking. This gap represents a promising area for future research, particularly given its potential to integrate school, family, and community interventions. By leveraging the combined influence of school environments and family dynamics, there is a significant opportunity to create sustainable improvements in the PA levels of children and their families.

This study has several limitations that should be acknowledged. Firstly, the recruitment of participants might have been biased towards more active families who were interested in the topic of PA. As a result, we may not be able to fully characterize the movement behaviors of families who declined to participate in the study, which could limit the generalizability of the findings. Secondly, there was a considerable dropout rate due to the requirement for a complete daily log. Many children did not have their logs filled out correctly or comprehensively, rendering their data unusable for analysis. This challenge could potentially be mitigated in future studies by replacing the paper-and-pencil approach with a digital solution, such as a mobile application that records GPS data. However, the use of such technology might also face resistance from participants due to privacy concerns and the requirement for informed consent. Thirdly, the study did not account for the participants' sports backgrounds, which could have provided important context for understanding their movement behaviors. Furthermore, a limitation of this study is the lack of information on whether all children from participating families were included and the absence of data on the number and ages of other children in these families. Fourthly, our final sample size was smaller than initially planned, which may reduce the statistical power to detect subtle effects. Nonetheless, the observed effects remained statistically significant, suggesting that key findings are robust. To reinforce generalizability and validate these results, future research with larger and more diverse samples is recommended. Finally, differences in school curricula systems and environmental conditions including the distance to school, were not considered, which may have influenced the children's PA patterns. Despite these limitations, the study provides valuable insights into the role of schools and families in shaping children's PA behaviors.

## Conclusion

In conclusion, the findings of the FAMIPASS study highlighted the need for schools to play a proactive role in promoting PA across multiple domains—through in-school activities, support for active commuting, and family engagement. Future interventions should aim at enhancing school-based PA programs while also extending support to families to ensure that active lifestyles are fostered both at school and at home. By leveraging the combined influence of school environments and family dynamics, there is a significant opportunity to create sustainable improvements in the PA levels of children and their families.

The segmentation of the school day revealed low activity levels before school, pointing to a critical area for intervention. Fathers' PA levels and education emerged as particularly strong influences on children's PA during and after school, while mothers' PA also played a positive role. These findings emphasize the importance of family-centered strategies that target both children and parents. Despite limitations such as selection bias, the study's use of synchronized accelerometer data provided robust insights into parent-child activity patterns, supporting the development of tailored, holistic approaches that integrate efforts from schools, families, and communities.

## Data Availability

The raw data supporting the conclusions of this article will be made available by the authors, without undue reservation.
